# Methylation, Hydroxylation, Glycosylation and Acylation Affect the Transport of Wine Anthocyanins in Caco-2 Cells

**DOI:** 10.3390/foods11233793

**Published:** 2022-11-24

**Authors:** Yang Liu, Jiali Lin, Tiantian Cheng, Yangjie Liu, Fuliang Han

**Affiliations:** 1 College of Enology, Northwest A&F University, Yangling, Xianyang 712100, China; 2 Shanxi Engineering Research Center for Viti-Viniculture, Northwest A&F University, Yangling, Xianyang 712100, China; 3 Heyang Experimental Demonstration Station, Northwest A&F University, Weinan 715300, China; 4 Ningxia Helan Mountain’s East Foothill Wine Experiment and Demonstration Station, Northwest A&F University, Yongning, Yinchuan 750104, China

**Keywords:** wine, anthocyanins, Caco-2 cell monolayer, absorption, SGLT1, GLUT2

## Abstract

Anthocyanins are substances with multiple physiological activities widely present in red wine, but the influence of structure (methylation, hydroxylation, acylation, glycosylation) on the transport remains ill-defined. In the present study, Caco-2 monolayers were used as an in vitro model of the absorptive intestinal epithelium to transport different types of anthocyanin samples. Results showed that both methylation and acetylation promote the level of transport. Monoglycoside standard exhibited higher transport amount and rate compared to diglycoside standard while the transport level of the monoglycoside mixture was unexpectedly lower than that of the diglycoside mixture. Caco-2 monolayers appeared to be more capable of transporting the single standard than the mixed standard. Meanwhile, the transport of anthocyanins in Caco-2 cell model showed time- and concentration-dependent trends. Anthocyanin treatment had a greater effect on sodium-dependent glucose transporter 1 (SGLT1) mRNA expression than glucose transporter 2 (GLUT2), and significantly down-regulated the protein expression of SGLT1. Although the low bioavailability of anthocyanins requires much more research, further evidence of the role of structure is provided.

## 1. Introduction

**Figure 1:**
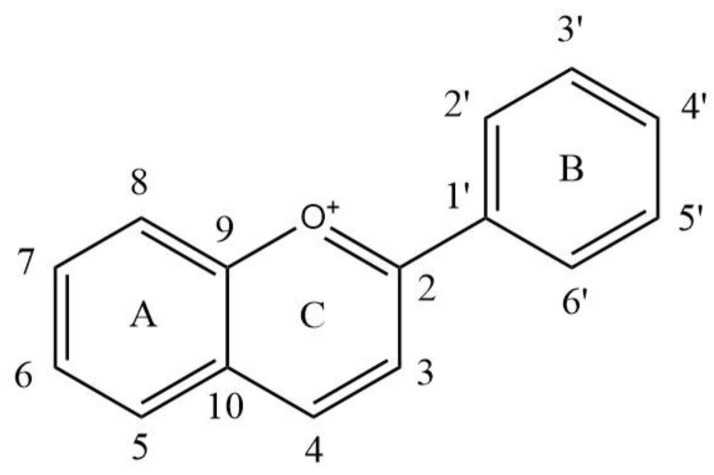
The basic structure of anthocyanins. A: A ring; B: B ring; C: C ring. 1–10 and 1′–6′: the number of the carbon atom.

Anthocyanins are natural flavonoid compounds formed by combining at least one glucose, galactose, rhamnose or arabinose molecules with the 3 or 5 hydroxyl group of the anthocyanidins C or A ring (Figure 1 and Table 1), as a water-soluble pigment, widely distributed in fruits, vegetables and corresponding foods or drinks [1,2]. The anthocyanins in wine are mainly derived from grapes. Grape varieties, cultivation and terroir affect the content and types of anthocyanins in grapes, and further in wine. In a full-bodied young red wine, the free anthocyanin content is generally 100–1000 mg/L, and it can reach 2000 mg/L in some cases [3,4]. Glycosylation, acylation, hydroxylation and methylation of anthocyanins are the main sources of structural diversity [5]. Anthocyanins monoglycosides dominated in young red wines made by *Vitis vinifera* grape varieties, such as Cabernet Sauvignon, rather than non-*Vitis vinifera* grape varieties in which anthocyanins diglycosides are the most abundant red pigments, such as *Vitis davidii* [3,6]. Thus, there are twelve basic anthocyanins in grape and wine, namely 6 anthocyanin monoglycosides (pelargonidin-3-*O*-glucoside (Pg-3-glc), delphinidin-3-*O*-glucoside (Dp-3-glc), cyanidin-3-*O*-glucoside (Cy-3-glc), petunidin-3-*O*-glucoside (Pt-3-glc), peonidin-3-*O*-glucoside (Pn-3-glc) and malvidin-3-*O*-glucoside (Mv-3-glc)) and 6 anthocyanin diglycosides (pelargonidin-3,5-*O*-diglucoside (Pg-3,5-diglc), delphinidin-3,5-*O*-diglucoside (Dp-3,5-diglc), cyanidin-3,5-*O*-diglucoside (Cy-3,5-diglc), petunidin-3,5-*O*-diglucoside (Pt-3,5-diglc), peonidin-3,5-*O*-diglucoside (Pn-3,5-diglc) and malvidin-3,5-*O*-diglucoside (Mv-3,5-diglc)) [7]. Mv-3-glc and Mv-3,5-diglc are the highest content of anthocyanins in *V. vinifera* and *V. davidii* young red wine, respectively.

The growing body of preclinical, clinical and observational evidence supports the potential physiologically active properties of anthocyanins and their derivatives, including anti-oxidation [2,5,8], anti-inflammatory [9,10,11], reducing the probability of cancer occurrence and recurrence [12], preventing and improving cardiovascular disease and type 2 diabetes [13,14], neuroprotection [15,16]. Few anthocyanins are digested by epithelial cells and bacteria, and most of them enter the stomach where anthocyanin glycosides were quickly and efficiently absorbed and rapidly excreted into intestinal tract as intact and metabolized forms [17]. However, the intestine is the main absorption site for anthocyanins. Two transporters, SGLT1 in the brush border and GLUT2 in the basal membrane of the small intestine epithelium, are involved in the absorption of anthocyanins from the intestinal apical into the cells, then passing through the basal side to the blood. SGLT1 is a Na^+^ and energy-dependent cotransporter, which can transport extracellular sodium ions and glucose into the cell at the same time. The transmembrane potential formed by the difference in Na^+^ concentration provides energy for the process [14,18], and GLUT2 helps the glucose in the intestinal cells to be discharged from the basal side into the blood without consuming energy [19]. It has been reported that GLUT2 may transfer from the basal side of the small intestine cells to the brush border helper cells for bulk quantities of glucose absorption through a special route when the contribution of the diffusive component to absorption exceeds that of the SGLT1-mediated component [20]. Although anthocyanins are beneficial to health, and the intake can easily reach 200 mg/d by eating fruits, vegetables and drinks rich in anthocyanins, the bioavailability of anthocyanins in the human body is very low (less than 1%) which closely related to its absorption level [21,22]. At the same time, the structure of anthocyanins influences its chemical properties and determines its stability and potential biological activity [13,23]. Additionally, the number of hydroxyl groups, acylation groups and glycosyl types all affect the polarity, size and space concept of a single compound, and also have a certain impact on the bioavailability [24,25].

Anthocyanin transport efficiency is closely related to its structure [13,26]. Oliveira et al. used MKN-28 gastric cells to compare the transport efficiency of anthocyanins with different structures. It was found that compared with the monoglycoside transport efficiency of methyl anthocyanin (about 10%), substitution with disaccharide and acylation (about 8–7%) obviously hindered the transport [27]. The transport efficiency of Dp-3-glc in blueberry extract is lower than that of Pn-3-glc and Mv-3-glc, which may be due to Dp-3-glc containing more hydroxyl groups and no methoxyl group, while Pn-3-glc contains one methoxyl group and Mv-3-glc contains two, suggesting that a decrease in hydrophilic groups and an increase in hydrophobic groups may facilitate the uptake of anthocyanins [28,29]. In situ perfusion studies revealed that the absorption rate of Mv-3-glc with a higher degree of B ring methylation was lower than that of Cy-3-glc, which was only 10.7%, while the latter reached 22.4% [30]. Studies have shown that anthocyanins with double substituents on the B ring have better transport effects. In NCI-N87 cell monolayers, Cy-3-glc and Pn-3-glc are preferentially transported and absorbed. When the aglycone structure is the same, the type of glycosyl may also affect the absorption of anthocyanins, and the transport rate of pentose glycosylated anthocyanins is higher than that of hexosylated anthocyanins [31]. Human trials indicate that acylation reduces the absorption efficiency of anthocyanins, and the absorption efficiency of anthocyanin-3-(xylose-spiroyl-glucose)-galactoside is lower than that of non-acylated anthocyanins with the same structure [32].

Therefore, the objectives of current work were to investigate the absorption of grape anthocyanin extracts and anthocyanin standards using Caco-2 cell monolayer model simulate the absorption process of the jejunum, and chemical structure on the bioavailability of different types of anthocyanins as well as the effect on mRNA and protein expression of two kinds of anthocyanin glucose transporter, SGLT1 [33] and GLUT2 [34]. This work can provide very useful information about anthocyanin bioavailability in *V. vinifera* and *V. davidii* young red wine in order to better understand the benefit of these anthocyanins.

## 2. Materials and Methods

### 2.1. Materials and Chemicals

Low Glucose Dulbecco’s Modified Eagle Medium (DMEM), GlutaMAX-1, Trypsin-EDTA and nonessential amino acids were purchased from Thermo Fisher Scientific Inc. (Shanghai, China). Penicillin-Streptomycin Solution, Thiazolyl Blue (MTT) and Hank’s Balanced Salt Solutions (HBSS) were purchased from Solarbio Scientific Ltd. (Beijing, China). Fetal Bovine Serum was purchased from Gemini Biotechnology Company (America). 2 × PLUS SYBR Green qPCR Mix kit was purchased from Nanjing Jiancheng Bioengineering Institute (Nanjing, China). Rabbit polyclonal antibodies (Anti-SGLT1 and Anti-GLUT2) were obtained from Bioss Biological Technology Co., Ltd. (Beijing, China). Mv-3-glc and Mv-3,5-diglc were purchased from Sigma-Aldrich, Inc. (St. Louis, MO, USA). Phloridzin and Phloretin were obtained from Aladdin Biochemical Technology Co., Ltd. (Shanghai, China).

### 2.2. Preparation of Anthocyanin Samples

Anthocyanin-rich extracts were prepared following methods of Han et al. (2020) [35], which we previously reported. Briefly, 5 g of grape skin was homogenized in 20 mL of hydrochloric acid: methanol (1:1000, *v*/*v*) at 40 °C for 30 min. Then, ultrasonic for 20 min, centrifuge at 8000 g/min for 5 min to collect the supernatant, the crude extract was filtered by 0.22 μm and purified by silica gel column. The organic phase eluate was collected and removed the solvent; the dried extract reconstituted in water was added with ethyl acetate to remove non-anthocyanin polyphenols. The anthocyanin was then purified by high-performance liquid chromatography with an analytical C18 column (LiChrospher RP-18 (Merck KGaA, Darmstadt, Germany), 250 × 4.0 mm, 5 μm) and the final anthocyanin extract was obtained after freeze-drying, which used for further analysis. The monosaccharide anthocyanin mixture was extracted from the Yan-73 grape, and the disaccharide anthocyanin mixture from *V. davidii*, which has been proven before [3,35]. All anthocyanin samples used in this study were dissolved in HBSS.

### 2.3. HPLC Analysis

The analysis of anthocyanins was based on the method published by Yang et al. [7] in 2018 with minor changes. Anthocyanins were detected by Shimadzu HPLC system (Shimadzu, Kyoto, Japan) equipped with LC-20AT pumps and a Synergi Hydro-RP C18 column (250 × 4.6 mm, 4 μm, Phenomenex, Torrance, CA, USA) to separate the anthocyanins in the samples under a flow rate of 1 mL/min. The wavelength on the photodiode array detector was set at 520 nm. The mobile phase A consisted of ultrapure water: acetonitrile: formic acid = 16:2:1 (*v*/*v*/*v*) and B ultrapure water: acetonitrile: formic acid = 8:10:1 (*v*/*v*/*v*). Elution procedure: 0–0.1 min: 0–5% B; 0.1–20 min: 5–100% B; 20–30 min: 100% B; 30–31 min: 100–5% B; 31–36 min: 5% B.

Mv-3-glc was used as the external anthocyanin standard for the quantitation of monoglycoside anthocyanins, and Mv-3,5-diglc was used for the diglycoside anthocyanins.

### 2.4. Cultivation of Caco-2 Cells and Cytotoxicity Tests

#### 2.4.1. Cultivation of Caco-2 Cells

Caco-2 cells were obtained from the Cell Bank of Fuheng Biological Technology Co., Ltd. (Shanghai, China) and incubated at 37 °C in a humidified atmosphere with 5% CO_2_ and 90% relative humidity and were cultivated in 15% of DMEM. The liquid was changed every day, and a Caco-2 cell monolayer model was formed after 17 days of culture, showing stable transmembrane resistance. Additionally, the cell monolayer had obvious polarity and no intercellular space, the microvilli were continuously and uniformly distributed on the cell surface, which could be used to simulate the absorption of substance in jejunum.

#### 2.4.2. Cytotoxicity Tests

The MTT assay was carried out to determine the survival of cells exposed to different concentrations of anthocyanins. Caco-2 cells with density of 1.5 × 10^5^ cells/mL (150 μL) were inoculated into 96-well plates per well and incubated 4 days to allow cells to sufficiently attach. After sucking out the culture medium, the cells were cultured for 4 h with or without 150 μL monoglycoside mixture, diglycoside mixture, monoglycoside standard, diglycoside standard and standard mixture of monoglycoside and diglycoside with concentrations of 400, 200, 100, 50, 25 μM, respectively, add 3 wells for each concentration. Three wells without cells were selected as the blank group, and 150 μL HBSS was added. Ten microliters of MTT (5 mg/mL) were added to each well and cultured for another 4 h. The absorbance was measured at 450 nm and the following formula was used to calculate the cell survival rate.

Cell survival rate (%) = (sample group OD value − blank group OD value)/(control group OD value − blank group OD value) × 100%

### 2.5. Transmembrane Transport of Anthocyanins in Caco-2 Cells

According to the results of cytotoxicity tests, anthocyanin solutions with concentrations of 100 and 200 μM were finally selected for transport experiments, and the two concentrations of monoglycoside mixture, diglycoside mixture and Mv-3-glc, Mv-3,5-diglc, Mv-3-glc + Mv-3,5-diglc three kinds of anthocyanin standard solutions, filtered through 0.22 μm nylon filters prior for use.

Each group selected 4 wells in the trans well chamber, 0.5 mL of the above anthocyanin solutions were added to the apical chamber and 1.5 mL sterile HBSS to the basolateral chamber. Samples were incubated at 37 °C, 5% CO_2_ and humidity 90%. Then, 150 μL was sampled from the basolateral chamber at 0.5 h, 1 h, 2 h and 4 h, stored at −20 °C until HPLC analysis.

### 2.6. Anthocyanin Stability Test

In order to investigate the stability of anthocyanins in a simulated cell transport environment, HBSS preheated at 37 °C was used to prepare a concentration of 200 μM five kinds of anthocyanin solutions (monoglycoside mixture, diglycoside mixture, monoglycoside standard, diglycoside standard and standard mixture of monoglycoside and diglycoside), each solution contained two replicates. Solutions were incubated at 37 °C, 5% CO_2_ and humidity 90%, samples were taken at 0 and 4 h, respectively, and stored at −20 °C until HPLC analysis.

### 2.7. Experiments on the Influence of Phloridzin and Phloretin on the Transport of Mv-3-glc

In order to verify whether SGLT1 and GLUT2 are involved in the absorption of Mv-3-glc in intestine, experiments influence of phloridzin (SGLT1 inhibitor [33]) and phloretin (GLUT2 inhibitor [34]) on the transport of Mv-3-glc were carried out. Phloridzin and phloretin were prepared in absolute ethanol at the stock concentration of 3 × 10^4^ μM, and then dispersed with HBSS into 300, 50, 10 μM for later use. After removal of the medium, the cells were washed three times with HBSS in 14 wells of the trans well chamber. Then, 0.25 mL 60 μg/μL Mv-3-glc solution was added to the apical chamber, and then mixed with 0.25 mL phloretin or phloridzin solution with the above three concentrations, respectively, while the basolateral chambers were refilled with 1.5 mL HBSS. The control group used 0.25 mL HBSS instead of phloridzin or phloretin solution. Samples were incubated in CO_2_ incubator, and 0.5 mL was sampled from the basolateral chamber at 30, 60, 120, 180 and 240 min to detect the content of Mv-3-glc, 0.5 mL HBSS was supplemented after each sample. All treatment conditions were conducted in duplicate.

### 2.8. RT-qPCR and Western Blotting Analysis

Total RNA was isolated from the cells samples before and after transfer using 2 × PLUS SYBR Green qPCR Mix kit (CWBiotech, Beijing, China) according to the manufacturer’s instructions. Expression levels of SGLT1 and GLUT2 mRNA were analyzed by real-time quantitative PCR using a Quant Studio 6 Sequence Detection System (Applied Biosystems, Waltham, MA, USA) and the reagent kit. The primer sequences used for each gene were given in Table 2, β-Actin was the internal reference gene. Quantitative measurements of transporters relative to GAPDH gene expression were derived using the 2^−ΔΔCT^ method.

Rapidly lysing the cells in radio immunoprecipitation assay (RIPA) buffer to collect the protein, protein expression of SGLT1 and GLUT2 was analyzed by Western blotting performed on the lysed cells, referred to Wong et al. [36]. For protein separation, the sample was adjusted in polyacrylamide gels for steady flow first, and then for electrophoresis under voltage stabilization until the loading buffer reached the bottom of the gel. Proteins were transferred to a PVDF membrane and incubated with the primary antibody and the HRP-labeled secondary antibody, respectively, then visualized with ECL Assay Kit (Monad Biotech Co., Ltd., Suzhou, China) enhanced chemiluminescence. The protein was visualized by ChemiDoc XRS+ Imaging System (Bio-Rad, Hercules, CA, USA) with Image Lab 3.0 software (Bio-Rad, Hercules, CA, USA).

### 2.9. Statistical Analysis

Except for explicitly stated, all data were expressed as mean ± standard deviation from three independent measurements. Figures were produced using Origin 2021 software (Origin Lab, Northampton, MA, USA). Differences were analyzed using the LSD analysis as appropriate with the help of SPSS 21.0 software (SPSS, Inc., Chicago, IL, USA). The statistical significance was assigned at *p* < 0.05.

## 3. Results and Discussion

### 3.1. Cytotoxicity Tests

After treatment with various concentrations of anthocyanin solutions for 4 h followed by the MTT assay, the survival rate of the corresponding cells was revealed in Table 3. Although 25 and 50 μM anthocyanin solutions have little effect on cell survival, they are not conducive to the detection of anthocyanin content in the sample after transportation, and 400 μM has a considerable degree of lethal effect on cells. In the end, the concentration 100 and 200 μM anthocyanin solutions were used for transport experiments. The cell survival rates at these concentrations were higher than 70%, which is conducive to further experiments.

### 3.2. Influence of Structure and Concentration on Transport

#### 3.2.1. The Effect of Structure and Concentration on the Transport of Anthocyanin Extracts in Caco-2 Cells

##### Transport of Monoglycoside Mixture

**Figure 2:**
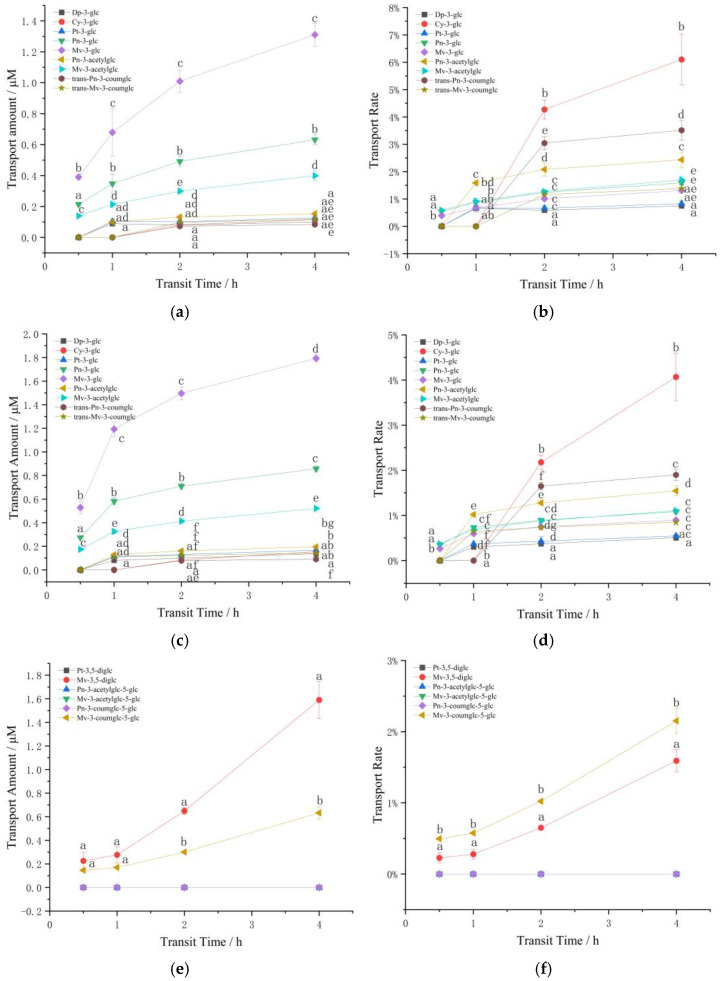

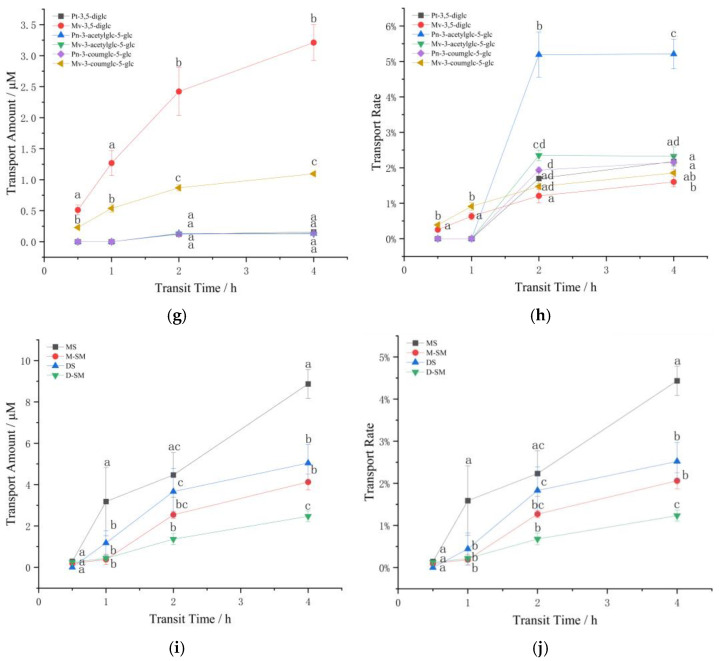
Changes in the transport amount and transport rate of the anthocyanins. (**a**) The transport amount of 100 μM monoglycoside mixture; (**b**) The transport rate of 100 μM monoglycoside mixture; (**c**) The transport amount of 200 μM monoglycoside mixture; (**d**) The transport rate of 200 μM monoglycoside mixture; (**e**) The transport amount of 100 μM disaccharide mixture; (**f**) The transport rate of 100 μM disaccharide mixture; (**g**) The transport amount of 200 μM disaccharide mixture; (**h**) The transport rate of 200 μM disaccharide mixture; (**i**) The transport amounts of standard anthocyanins; (**j**) The transport rate of standard anthocyanins. Different small letters indicate significant difference at *p* < 0.05 in each figure. MS: monoglycoside standard; DS: diglycoside standard; M-: the transport information of monoglycosides; D-: the transport information of diglycosides; SM: standard mixture of monoglycoside and diglycoside.

Nine kinds of anthocyanins were detected by HPLC in the samples after the transport of monoglycoside mixture, including five basic anthocyanins (Dp-3-glc, Cy-3-glc, Pt-3-glc, Pn-3-glc and Mv-3-glc), two acetylated anthocyanins (peonidin 3-*O*-(6-*O*-acetyl)-glucoside (Pn-3-acetylglc) and malvidin-3-*O*-(6-*O*-acetyl)-glucoside (Mv-3-acetylglc)) and two coumaroylated anthocyanins (peonidin 3-*O*-(6-*O*-p-coumaroyl)-glucoside (Pn-3-coumglc) and malvidin-3-*O*-(6-*O*-p-coumaroyl)-glucoside (Mv-3-coumglc)). Figure 2a–d reveals the changes in the transport amount and transport rate of the monosaccharide anthocyanin mixture in the Caco-2 model over 4 h.

Figure 2a,c indicated that the transport amount of trans-Pn-3-coumglc was lowest. Dp-3-glc, Cy-3-glc and Pt-3-glc were not detected at 0.5 h, and prolonging the transport time can effectively improve the transport level of anthocyanins, time-dependent on the transport of monosaccharide anthocyanin extracts existed in Caco-2 model, the longer the transfer time, the higher the anthocyanin transfer amount and transfer rate, which also demonstrates that the transporter may have a period of adaptation to anthocyanins. Meanwhile, concentration effect also appeared, the higher the concentration, the greater the transport amount. It is worth noting that the transport of anthocyanins is not simply increased in proportion to the increase in concentration. In addition, the transport of most anthocyanins first increased rapidly and then gradually slowed down, suggesting that cells may exist a saturation effect on the transport of anthocyanins, relating to active transport involving transporters.

A comparison of the transfer rates of anthocyanins at different concentrations showed that the transport rate of Mv-3-glc > Pt-3-glc > Dp-3-glc, which is the higher the methylation degree of anthocyanin, the lower the hydroxylation degree, the higher the transport rate. Simultaneously, the transport rate of coumarin acylation > acetylation > unacylated, and the difference was significant (*p* < 0.05). This may be due to acylation increasing the steric hindrance of the binding of anthocyanins to the transporter [37]. While for Mv-3-glc, no significant difference was observed between the two acylated and the unacylated forms. Nonetheless, the methylation degree of Mv-3-glc was higher than that of Pn-3-glc and its transport efficiency was low with the same structure of acetylation, which may be due to the increase in the number of methoxy groups in the B ring, which is beneficial to the stability of the anthocyanin structure [7]. All anthocyanins reached the maximum transfer rate when the transfer time was 4 h. Interestingly, all anthocyanins performed higher transfer rates at lower concentrations, which indicated that there may be an optimal concentration of transferred anthocyanins. Too high concentration is not conducive to its transportation.

##### Transport of Diglycoside Mixture

Only 6 types of anthocyanins were detected in the diglycoside mixture after being transported by the Caco-2 cell model, which were two basic anthocyanins (Pt-3,5-diglc and Mv-3,5-diglc), two acetylated anthocyanins (peonidin-3-*O*-(6-*O*-acetyl)-glucoside-5-*O*-glucoside (Pn-3-acetylglc-5-glc) and malvidin-3-*O*-(6-*O*-acetyl)-glucoside-5-*O*-glucoside (Mv-3-acetylglc-5-glc)) and two coumaroylated anthocyanins (peonidin-3-*O*-(6-*O*-p-coumaroyl)-glucoside-5-*O*-glucoside (Pn-3-coumglc-5-glc) and malvidin-3-*O*-(6-*O*-trans-p-coumaroyl)-glucoside-5-*O*-glucoside (Mv-3-coumglc-5-glc)).

As can be seen from Figure 2e–h, Mv-3,5-diglc exhibited the highest transport amount at the two concentrations (1.59 and 3.21 μM, respectively), followed by Mv-3-coumglc-5-glc (0.63 and 1.10 μM, respectively) after 4 h. Accordingly, the transfer amount of high-concentration diglycoside mixture significantly higher than that of low-concentration, and Pt-3,5-diglc, Pn-3-acetylglc-5-glc, Mv-3-acetylglc-5-glc, Pn-3-coumglc-5-glc were only detected at high concentrations after 2 h of transport, and suggested a concentration dependence between 2–4 h.

Comparisons of transport rates were shown in Figure 2f,h. The transport rate of Pt-3,5-diglc was higher than Mv-3,5-diglc, the higher the degree of B-ring methylation, the lower the transport rate, which is consistent with the monosaccharide mixture. Furthermore, although Mv-3,5-diglc presented the highest transport amount and the transport rate was almost three times that of Mv-3-coumglc-5-glc, the transport speed of Mv-3-coumglc-5-glc at low concentration was significantly higher than that of Mv-3,5-diglc. At high concentration, the transport rate of Mv-3-coumglc-5-glc was higher than that of Mv-3,5-diglc, and the transport rate of Mv-3-acetylglc-5-glc was also higher than that of Mv-3-coumglc-5-glc. This exhibited the same rule as that of monosaccharide mixtures: the transport rate of acylated anthocyanins was higher than unacylated, but the transport rate of acetylated anthocyanins is higher than that of coumaroylated anthocyanins in disaccharide mixtures. This also occurred at high concentrations of Pn-3-acetylglc-5-glc and Pn-3-coumglc-5-glc transport, this may be related to the larger structure of coumarin and the increased number of glycosides. At the same time, the transport rate of the same acylated anthocyanins showed the same law at higher concentrations, Mv-3-coumglc-5-glc < Pn-3-coumglc-5-glc, Mv-3-acetylglc-5-glc < Pn-3-acetylglc-5-glc, that is, the higher degree of methoxylation, the lower the disglycoside transport rate, which is consistent with the situation in the monosaccharide mixture.

Notably, even though a certain anthocyanin exhibits the highest amount of transport, its transport rate may be the lowest, which may signal that the additional mechanisms of anthocyanins uptake and transport [31].

#### 3.2.2. The Effect of Structure and Concentration on the Transport of Standard Anthocyanins in Caco-2

Considering the concentration effect of anthocyanin transport, only higher concentration of 200 μM was selected for the transport of anthocyanin standard. Figure 2i,j illustrated the effect of structure and concentration on the transport of standard anthocyanins in Caco-2. Similar to Oliveira’s results [27], significantly higher transshipment amount was found between Mv-3-glc and Mv-3,5-diglc, and occurred simultaneously in the transport of single standard and mixed standard of anthocyanins, we speculated that the anthocyanin transport rate decreases significantly with the increase of the number of glucose molecules, that is to say the disaccharide structure is not conducive to the transport of anthocyanins. In the meantime, Mv-3-glc and Mv-3,5-diglc both showed higher transport amount when transported separately at 4 h (8.87 and 5.04 μM), which was higher than that of the mixed standard (4.12 and 2.47 µM), and this likely reflects the limited number of transporters.

Similar to the results of transshipment, the single glycoside standard Mv-3-glc showed the highest transshipment rate in the single standard, followed by the single standard Mv-3,5-diglc. Meanwhile, except for single standard Mv-3-glc, all the other anthocyanins performed the highest transport rate at 1–2 h after transport, suggesting that there may be an adaptation mechanism for the transport of anthocyanins in Caco-2 cells. At the initial stage of transport, anthocyanins stimulate the cells to transport and absorb them, and this promoting effect decreases with the extension of contact time.

### 3.3. The Influence of Simulated Cell Transport Conditions on the Stability of Anthocyanins

**Figure 3:**
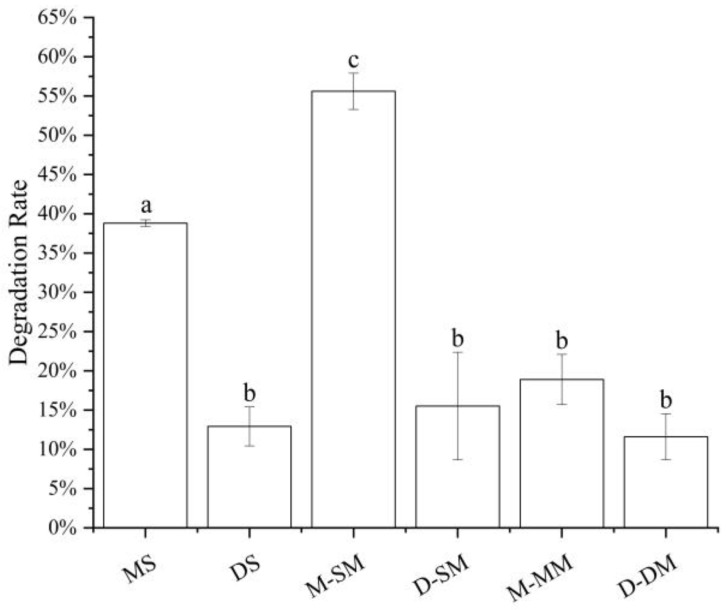
Anthocyanins degradation rates under simulated cell transport conditions. Different small letters indicate significant difference at *p* < 0.05 in each figure. MS: monoglycoside standard; DS: diglycoside standard; M-: the transport information of monoglycosides; D-: the transport information of diglycosides; SM: standard mixture of monoglycoside and diglycoside; MM: monoglycoside mixture; DM: diglycoside mixture.

Anthocyanins are more stable in acidic than those in alkaline media [2,7,21]. When the pH is lower than 3, anthocyanins exist in the form of red and yellow sulfonate cations, while in neutral HBSS solution, anthocyanins are affected by the affinity of water molecules. Nuclear attack on the C2 position transfers the protons of the hydroxyl group, and the anthocyanins are degraded by ring-opening to form chalcone [1,23,25,38]. Therefore, the degradation and stability of anthocyanin itself may also be the reason that affects the transport effect of anthocyanin in different samples. It can be seen from Figure 3 that in all samples, monoglycosides performed a higher degradation rate than diglycosides, and whether it is monoglycosides or diglycosides, the stability of the mixtures was higher than single standard anthocyanins, and higher than that in the mixed standards. The better stability may be the reason that the diglycoside mixture was higher than the monoglycoside mixture and the single standard higher than the mixed standard in terms of transport amount and transport rate, but this does not apply to the monoglycoside and diglycoside single standards transshipment. The difference in stability could be affected by the structure of anthocyanins.

### 3.4. The Effect of Phloridzin and Phloretin on the Transport of Mv-3-glc

**Figure 4:**
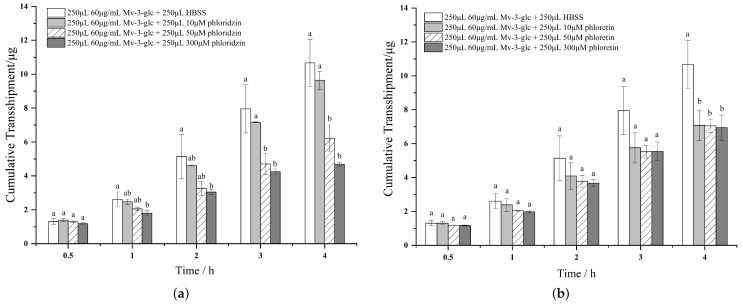
Effects of phloridzin (**a**) and phloretin (**b**) on the cumulative transfer of Mv-3-glc. Different small letters indicate significant difference at *p* < 0.05 in each figure.

Compared with the control group, both the three concentrations of phloridzin reduced the cumulative transport of Mv-3-glc, and the longer the time, the more significant the inhibitory effect, and the higher the concentration, the less the transport (Figure 4). By contrast, the inhibitory effect of phloridzin at 10 μM was not significant (reduction of 9.56% in 4 h), and when the concentration reaches 50 μM, the transport amount can be significantly reduced in 3 h, and 41.63% in 4 h, 300 μM can significantly inhibit the transport amount at 1 h, and decrease by 56.13% at 4 h. However, for phloretin, the three concentrations also reduced the cumulative transport amount of Mv-3-glc, but the difference was statistically significant only at 4 h, when the transport amount decreased by 33.57%, 33.87% and 34.89% compared with the control group, respectively.

In summary, the above results confirmed that the absorption mode of Mv-3-glc monomer from the apical side to the basal side was involved by SGLT1 and GLUT2 carriers.

### 3.5. The Effect of Anthocyanin Transport on Cellular mRNA and Protein Expression

#### 3.5.1. The Effect of Anthocyanin Transport on Cellular mRNA

**Figure 5:**
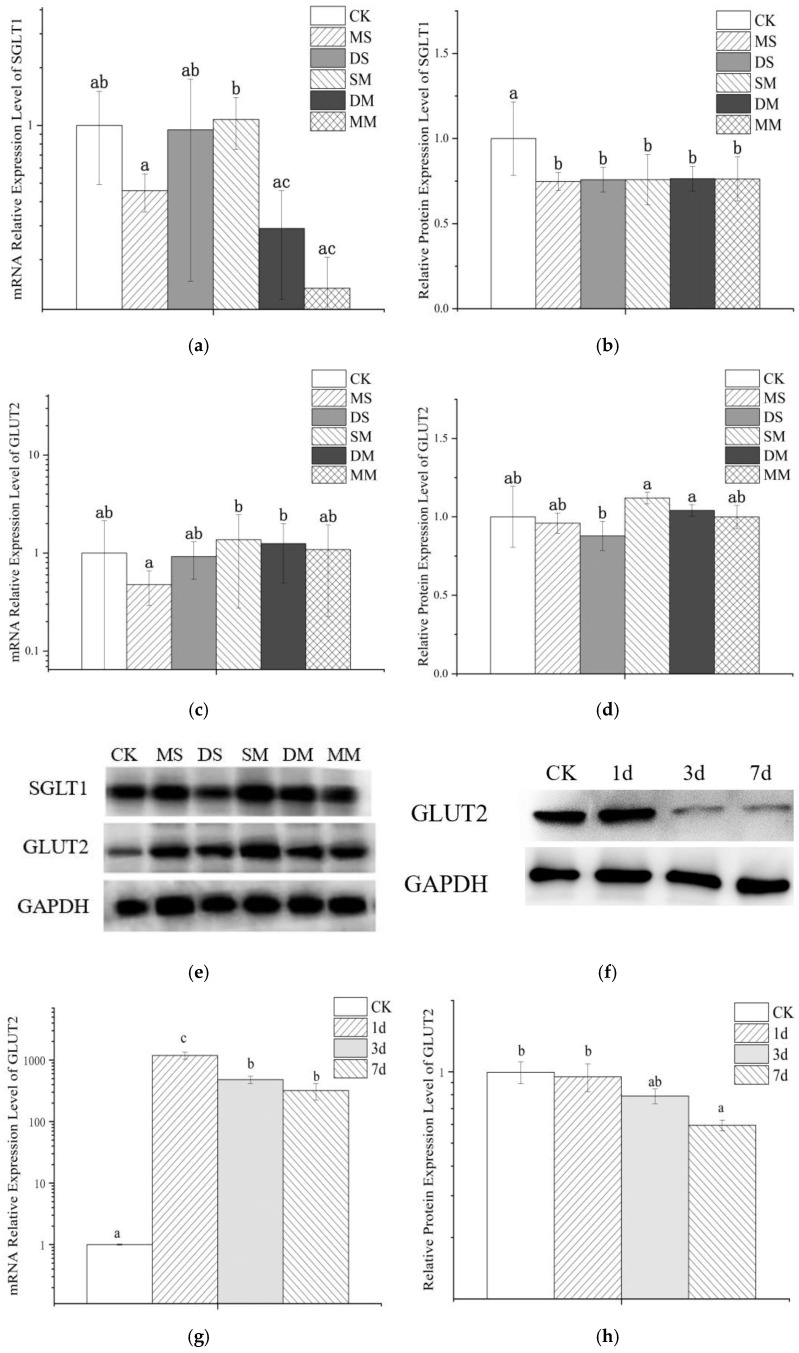
Effects of anthocyanins on mRNA and the expression of SGLT1 and GLUT2 during transport. (**a**) mRNA relative expression level of SGLT1; (**b**) relative protein expression level of SGLT1; (**c**) mRNA relative expression level of GLUT2; (**d**) relative protein expression level of GLUT2; (**e**) Western blotting of glucose transporter protein and internal reference protein; (**f**) Western blotting of GLUT2 and internal reference protein; (**g**) effects of anthocyanin pretreatment on GLUT2 mRNA; (**h**) effects of anthocyanin pretreatment on the relative protein expression of GLUT2. Different small letters indicate significant difference at *p* < 0.05 in each figure. CK: the control group; MS: monoglycoside standard; DS: diglycoside standard; SM: standard mixture of monoglycoside and diglycoside; DM: diglycoside mixture; MM: monoglycoside mixture; 1d: Mv-3-glc standard pretreatment for 1 day; 3d: Mv-3-glc standard pretreatment for 3 days; 7d: Mv-3-glc standard pretreatment for 7days.

Figure 5a,c exhibited the effect of anthocyanin transport on mRNA expressions of glucose transporters SGLT1 and GLUT2 in Caco-2 cell model. It can be observed that the monoglycoside mixture and the disaccharide mixture inhibit the expression of SGLT1 mRNA to a greater extent and slightly promote the expression of GLUT2 mRNA, while the monoglycoside standard can simultaneously hold-up the expression of SGLT1 and GLUT2 mRNA. At the same time, the relative expression of SGLT1 and GLUT2 mRNA increased after transporting the mixed standard, although this increase was not significant. Compared with GLUT2, wine anthocyanins had a greater effect on SGLT1 mRNA expression. Although there was no significant difference between the relative expression levels of SGLT1 and GLUT2 mRNA after transporting Mv-3-glc and Mv-3,5-diglc, respectively, the relative expression levels of the latter were larger. The relative expression of transporter mRNA in the mixed anthocyanin standard group was higher than that of the transporter mRNA when one of the standards was used alone, and the expression level was significantly higher than that after monoglycoside standard transport, indicating that the anthocyanin mixture had no effect on cells. The stimulatory regulation effect may be stronger.

#### 3.5.2. The Effect of Anthocyanin on Cell Protein Expression

Analysis of protein expression found that anthocyanins treatment significantly down-regulated the expression of SGLT1 protein in the Caco-2 cell model, but there was no significant difference between different groups. For GLUT2, no significant differences were observed among the GLUT2 protein expression in the Caco-2 cell model before and after anthocyanin transport. However, the mixed standard and diglycoside mixture treatment had a certain promotion effect on the protein expression of GLUT2, and diglycoside both single standard and monoglycoside single standard inhibited the expression of GLUT2 to varying degrees, and the impact of diglycoside was greater than that of monoglycoside. Furthermore, the influence of diglycoside mixture on the protein expression of GLUT2 was larger than that of monoglycoside mixture. Therefore, the carbohydrate cardinality and species richness of anthocyanins may be one of the factors affecting GLUT2 expression. The unsynchronized changes of protein and mRNA may be related to the combined results of translation and protein degradation [39], which further illustrated the complexity of the transcription and translation process.

In order to explore whether early exposure to anthocyanins would affect the transport of wine anthocyanins in the cell model, we selected some cells and used Mv-3-glc standard at a concentration of 22 mg/L for them during the culture process. 1d, 3d and 7d of preprocessing were carried out, respectively. It can be seen from Figure 5f–h that pretreatment significantly up-regulated the relative gene expression of GLUT2 in Caco-2 cells. Additionally, the expression of GLUT2 mRNA of 1d was significantly higher than that of 3d and 7d, and with the prolongation of preprocessing time, although the expression of GLUT2 mRNA decreased, the up-regulation was still significantly higher than that in the control group, which similar to the findings of Zhang et al., that shorter incubation (30 min) was more effective than longer exposure [40]. Meanwhile, Mv-3-glc pretreatment inhibited the relative protein expression level of GLUT2, and the effect enhanced with time. This suggested that long-term exposure to anthocyanins may have a certain inhibitory effect on glucose transporters in small intestinal epithelial cells, which may be the reason why the transport rate of wine anthocyanins decreases with the prolongation of pretreatment time.

Therefore, during the transport process, anthocyanins not only interact with the transporter, but also affect the transport by interfering with the expression of the transporter. Existing molecular docking analysis also showed that anthocyanins can directly bind with docking pocket of SGLT-1 and GLUT2, but a higher affinity for SGLT1 and a slightly higher surface complementarity between SGLT1 and anthocyanins [40].

## 4. Conclusions

In general, methylation, hydroxylation, glycosylation and acylation all impact the transport of wine anthocyanins in Caco-2 cells. Monoglycoside standard had a tendency to be transported more rapidly in caco-2 monolayers than that of diglycoside standard, while the amount and rate of the diglycoside mixture was higher than monoglycoside mixture, methoxy substitution on the B ring of anthocyanins inhibited the rate of transport. Meanwhile, anthocyanins were transported in a time- and concentration-dependent manner. Compared with GLUT2, the effect of wine anthocyanin on SGLT1 mRNA expression was more obvious. Anthocyanin treatment also significantly down-regulated the expression of SGLT1 protein in Caco-2 cell model, but GLUT2 did not. Furthermore, anthocyanins pretreatment and the stability of anthocyanins themselves are also the reasons for the differences in the transport levels of different samples. Therefore, the mechanism of the structure-induced transport difference of anthocyanins in Caco-2 cells needs to be further verified in future experiments.

## Figures and Tables

**Table 1:** Substitution of common anthocyanins.

Anthocyanins	Substitution Pattern						
	3	5	6	7	3′	4′	5′
Delphinidin (Dp)	OH	OH	H	OH	OH	OH	OH
Cyanidin (Cy)	OH	OH	H	OH	H	OH	H
Petunidin (Pt)	OH	OH	H	OH	OMe	OH	OH
Peonidin (Pn)	OH	OH	H	OH	OMe	OH	H
Malvidin (Mv)	OH	OH	H	OH	OMe	OH	OMe
Pelargonidin (Pg)	OH	OH	H	OH	H	OH	H

**Table 2:** qPCR reaction primers (5′–3′).

Gene	Primer	
	Forward Primer	Reverse Primer
Human SLC5A1 (AF070544.1)	CAGATGATGCGGGAGAAGAA	CGAAGATGCTCGTGGAGTAATA
Human SLC2A2 (J03810.1)	ATGAACTGCCCACAATCTCATA	GGACCAGAGCATGGTGATTAG
Human β-Actin (NM_001101.5)	CCTTCCTGGGCATGGAGTC	TGATCTTCATTGTGCTGGGTG

**Table 3:** Cell survival rate.

Concentration (μM)	Cell Survival Rate (%)				
	MM	DM	MS	DS	SM
0	100 ± 3.78 ^a,b^	100 ± 3.78 ^b^	100 ± 3.78 ^b^	100 ± 3.78 ^a,b^	100 ± 3.78 ^a^
25	118.57 ± 5.66 ^a^	103.00 ± 1.38 ^a^	117.14 ± 8.83 ^a^	108.14 ± 13.10 ^a^	95.00 ± 20.54 ^a^
50	100.71 ± 3.74 ^a,b^	99.29 ± 3.16 ^b^	95.71 ± 8.83 ^b^	93.71 ± 8.79 ^a,b^	92.57 ± 2.39 ^a,b^
100	84.00 ± 8.60 ^b^	92.71 ± 1.93 ^c^	86.57 ± 5.60 ^c^	88.29 ± 16.97 ^b,c^	81.43 ± 7.13 ^b,c^
200	81.71 ± 7.96 ^c^	85.71 ± 3.93 ^c^	86.29 ± 5.43 ^c^	75.29 ± 8.24 ^c^	73.71 ± 4.52 ^c^
400	63.71 ± 6.93 ^d^	46.71 ± 9.65 ^d^	74.71 ± 5.94 ^d^	57.57 ± 20.85 ^d^	52.43 ± 3.44 ^d^

Values are shown as the mean ± SD (n = 3). Different small letters indicate significant difference at *p* < 0.05. MM: monoglycoside mixture; DM: diglycoside mixture; MS: monoglycoside standard; DS: diglycoside standard; SM: standard mixture of monoglycoside and diglycoside.

## Data Availability

The data presented in this study are available on request from the corresponding author.
